# The Deep Head of the Masseter Muscle: A Classification-Based Anatomical and Surgical Framework

**DOI:** 10.3390/biomedicines13092201

**Published:** 2025-09-08

**Authors:** Adrian Okoń, Ingrid C. Landfald, Łukasz Olewnik

**Affiliations:** 1 Department of Anatomical Dissection and Plastination, Mazovian Academy in Płock, 09-402 Płock, Poland; a.okon@mazowiecka.edu.pl; 2 Department of Clinical Anatomy, Mazovian Academy in Płock, Plac Dąbrowskiego 2, 09-402 Płock, Poland; ingridceciliee@gmail.com; 3 VARIANTIS Research Laboratory, Department of Clinical Anatomy, Mazovian Academy in Płock, 09-402 Płock, Poland

**Keywords:** deep head, masseter muscle, anatomical variation, imaging, surgical anatomy, morphological classification, MRI, ultrasound, facial surgery

## Abstract

**Background:** The deep head of the masseter muscle (DHMM) is an underrecognized anatomical structure, frequently absent from standard anatomical references and often overlooked in maxillofacial surgical planning. Its morphological variability, spatial complexity, and relationship with neurovascular structures carry significant implications for imaging interpretation, diagnosis, and surgical outcomes. **Objective:** The objective of this paper is to synthesize current anatomical, embryological, and radiological knowledge on the DHMM, and to introduce a refined morphological classification with direct clinical and surgical relevance. **Methods:** A comprehensive literature review was performed, incorporating cadaveric dissections, radiological imaging (MRI, DTI, HRUS, CT), and clinical case reports. Emphasis was placed on anatomical variability, radiological detectability, and surgical accessibility. Based on these findings, a three-type classification with clinically relevant subtypes was formulated and correlated with imaging features and procedural risk. **Results:** The DHMM can be categorized into three principal types: Type I—classical form with fascial separation; Type II—fused with the medial pterygoid; Type III—segmented into two muscular bellies. Each type may present a **subtype b**, characterized by neurovascular penetration, which significantly increases surgical risk and alters procedural strategy. MRI and high-resolution ultrasonography were identified as the most reliable modalities for in vivo differentiation, with HRUS providing additional value for dynamic and volumetric assessment. **Conclusions:** Recognition of DHMM morphology, including high-risk neurovascular subtypes, is essential for accurate diagnosis, surgical planning, and prevention of complications. The proposed classification offers a reproducible framework for imaging standardization, surgical risk stratification, and integration into anatomical atlases and clinical guidelines.

## 1. Introduction

### 1.1. Classical Division of the Masseter Muscle: Superficial and Deep Heads

The masseter muscle (MM), a key component of the masticatory apparatus, has traditionally been described as comprising two primary layers: a superficial head (SHMM) and a deep head (DHMM). This classical bipartite division, originally emphasized by Belser et al. [1], is widely accepted and featured in standard anatomical textbooks, including Gray’s Anatomy [2]. The SHMM originates from the zygomatic arch and inserts into the lateral surface of the mandibular ramus, whereas the DHMM is typically located more posteriorly and inserts higher along the ramus and the coronoid process. However, this superficial–deep dichotomy has limitations when applied to individual anatomical variability, especially in the context of advanced imaging and surgical planning.

### 1.2. Lack of Consistent Criteria Defining the Deep Head

Despite its routine mention in the anatomical literature, the DHMM remains inconsistently described in terms of its origin, extent, and relationships with adjacent structures. The current definitions vary across studies, with some sources treating the DHMM as a continuation of the superficial layer, while others describe it as a distinct muscular compartment, sometimes even containing accessory bellies or neurovascular penetrations [3,4]. Moreover, the interface between the DHMM and the medial pterygoid muscle raises further ambiguity, blurring the morphological boundaries between these muscles. This lack of clarity impedes the establishment of a standardized anatomical classification.

### 1.3. Functional and Surgical Importance of the Deep Head

The DHMM plays a critical yet underrecognized role in mandibular biomechanics. Electromyographic and morphometric data suggest that this layer may contribute significantly to vertical bite force, as well as to medial and posterior stabilization of the mandible [5,6]. Clinically, the DHMM is of high relevance in orthognathic and oncological procedures involving the ramus or the parotid region. Anatomical misinterpretation of this region can lead to iatrogenic injury or misdiagnosis of mass-like lesions [7]. In aesthetic surgery, particularly in procedures involving botulinum toxin injections or selective myotomy, precise localization of the deep portion is crucial for predictable outcomes [8,9,10]. From a biomechanical standpoint, the DHMM may serve a distinct role compared to its superficial counterpart. While the SHMM contributes to elevation and slight protrusion of the mandible through its oblique fiber orientation, the DHMM, with its predominantly vertical fibers inserting on the superior ramus and coronoid process, appears to function primarily as a stabilizer during intercuspal biting and mandibular clenching [6]. Its deeper positioning and denser fiber architecture suggest a greater capacity for vertical force generation, particularly in high-resistance mastication or bruxism-related activity [5]. These mechanical distinctions further support the need to consider the DHMM as a functionally independent unit in both diagnosis and treatment planning.

### 1.4. Aims of the Present Review

This review aims to provide a comprehensive overview of the morphology and anatomical variability of the DHMM establish a novel morphological classification based on cadaveric and radiological data, correlate anatomical findings with imaging characteristics using ultrasound (US), magnetic resonance imaging (MRI), and computed tomography (CT), and outline the clinical and surgical implications of DHMM variants in various fields including maxillofacial surgery, oncology, and aesthetic medicine. By integrating anatomical, radiological, and clinical perspectives, we intend to highlight the need for reclassification of this anatomically and surgically significant structure.

## 2. Methods

This narrative review followed a structured literature search to synthesize anatomical and clinical knowledge regarding the DHMM. Searches were conducted in PubMed, Scopus, and Web of Science from database inception to 14 August 2025 (final search date).

Search strategy (example for PubMed): n “masseter muscle” AND (“deep head” OR “deep part” OR “deep layer”) AND (“anatomy” OR “morphology” OR “variation” OR “imaging” OR “MRI” OR “ultrasonography” OR “CT” OR “DTI”). Equivalent Boolean strings were applied in Scopus and Web of Science. Reference lists of included studies were hand-searched for additional articles. No language limit was imposed at the search stage; however, only records providing sufficient anatomical or clinical detail in English were included in the synthesis.

Inclusion criteria: (1) cadaveric studies describing DHMM morphology, attachments, innervation, vascularization, or anatomical variation; (2) imaging studies (MRI, CT, US, DTI) reporting DHMM identification/characterization; (3) clinical studies linking DHMM anatomy to surgical, aesthetic, or functional outcomes; (4) embryological/developmental studies relevant to the masseter complex.

Exclusion criteria: (1) non-human studies (unless used for comparative anatomy); (2) reports lacking primary anatomical or imaging data; (3) duplicates or conference abstracts without full text. Study selection and data extraction: Two authors independently screened titles/abstracts and then full texts; disagreements were resolved by consensus. Extracted data were organized into five domains: (1) gross anatomy and morphological variants; (2) embryology and development; (3) imaging features; (4) biomechanical/functional aspects; and (5) clinical and surgical implications. Classification development: The proposed three-type, two-subtype taxonomy was derived by qualitative synthesis of morphological descriptors abstracted from the included sources. Candidate features were grouped by structural similarity (presence of a fascial plane between superficial and deep heads; partial/complete fusion with the medial pterygoid; segmented/bipartite deep head). Neurovascular penetration was treated as an orthogonal attribute and encoded as subtype “b” within each type. Because the primary reports used heterogeneous measurement protocols, no prespecified quantitative cut-offs were imposed; when available, numeric ranges were recorded but not used to define thresholds. Two anatomists independently mapped features to provisional categories; disagreements were resolved by consensus. This exercise targeted face and content validity, not reliability testing.

Scope of evidence: This article is a narrative review; no new cadaveric dissections or original imaging datasets were generated for this work. Findings and the proposed classification are derived from the previously published literature. No formal inter-observer reliability testing or quantitative threshold validation was performed, as the present work aimed to establish a conceptual framework based on descriptive synthesis. These aspects are identified as priorities for future prospective imaging and cadaveric studies. Terminological inconsistencies across primary reports were reconciled by cross-referencing descriptions with established anatomical nomenclature (Terminologia Anatomica) and by standardizing variant definitions before inclusion in the classification framework.

No generative AI tools were used for content drafting, data generation, analysis, or scientific interpretation in this manuscript.

## 3. Embryological and Topographical Background

### 3.1. Development of First Pharyngeal Arch Musculature

The MM, along with the temporalis, medial pterygoid, and lateral pterygoid muscles, derives embryologically from the mesenchyme of the first pharyngeal (branchial) arch [11,12,13,14]. Myogenic progenitor cells migrate from the cranial paraxial mesoderm and undergo myogenic specification under the influence of transcription factors such as *PAX3*, *MYOD*, and *Tbx1*. These myoblasts aggregate into distinct muscle masses that eventually differentiate into individual masticatory muscles. By the 8th week of gestation, the muscle primordia of the MM are identifiable, with initial indistinct separation from the adjacent medial pterygoid and temporalis masses [15]. The DHMM is presumed to emerge from the posterior portion of this common mesenchymal field.

### 3.2. Separation of the Deep Head from the Medial Pterygoid Muscle: Morphogenetic Controversies

A long-standing debate exists regarding whether the DHMM should be considered a fully independent anatomical unit or a derivative of the medial pterygoid complex. Some developmental studies and dissections suggest that the medial pterygoid and DHMM share a common anlage during early morphogenesis, only later becoming distinguishable due to spatial rearrangement and directional growth [3]. In some specimens, fibromuscular continuity between the two structures has been observed, raising questions about the ontogenetic boundaries [16]. These findings complicate surgical dissection in the pterygomasseteric sling region, where fascial planes may be ambiguous or absent.

### 3.3. Vascular and Neural Variants: The Course of the Masseteric Nerve

The MM is predominantly innervated by the masseteric nerve, a branch of the anterior division of the mandibular nerve (V3), which typically passes through the mandibular notch alongside the masseteric artery [17]. However, the course of the masseteric nerve in relation to the DHMM is highly variable. In some cases, it penetrates directly into the deep portion before branching into superficial fibers, while in others it bifurcates outside the muscle and enters both heads independently [17,18]. Vascular variations are equally significant; the masseteric artery may give rise to branches that traverse or even perforate the DHMM [19], posing a potential risk during orthognathic surgery or parotidectomy.

### 3.4. Hypothesis of Pterygoid Origin: Histological and Ontogenetic Evidence

Based on topographical relationships and shared embryological origins, some authors have proposed that the deep head of the MM may represent a specialized, laterally displaced subdivision of the medial pterygoid muscle [3,16]. Histological studies have revealed similarities in fiber type composition and spindle distribution between these two muscles [4,20]. Furthermore, in certain primates and fetal human specimens, transitional muscle bundles can be observed bridging the medial pterygoid and deep masseter compartments, supporting the concept of a functional continuum [21]. Despite these findings, most anatomical texts maintain their distinct classification due to differences in orientation, insertion, and neurovascular supply.

## 4. Gross Anatomy and Variants

### 4.1. Classical Organization of the Superficial and Deep Heads of the Masseter Muscle

The MM is classically described as a quadrilateral muscle composed of two main layers: a SHMM and a DHMM, both arising from the zygomatic arch but differing in orientation, fiber arrangement, and insertion points [1,2]. The SHMM originates from the anterior two-thirds of the lower border of the zygomatic arch and inserts into the angle and inferior half of the lateral surface of the ramus of the mandible. It consists predominantly of oblique fibers running inferoposteriorly.

The DHMM, on the other hand, arises from the inner surface and posterior third of the zygomatic arch and projects more vertically to insert along the upper half of the mandibular ramus and sometimes extends toward the coronoid process [3,4]. This layer is thought to contribute to vertical force generation and stabilization of the temporomandibular joint (TMJ), particularly during powerful masticatory activity [6].

Macroscopic dissections reveal that the DHMM lies medial and posterior to the superficial layer, often separated by a distinct fascial septum, although in some individuals this fascial boundary is attenuated or absent, resulting in partial fusion or blending of fibers between the two layers [22]. This overlap contributes to difficulties in both anatomical dissection and radiological differentiation, especially in patients with hypertrophic or asymmetric MM’s [7].

**Figure 1:**
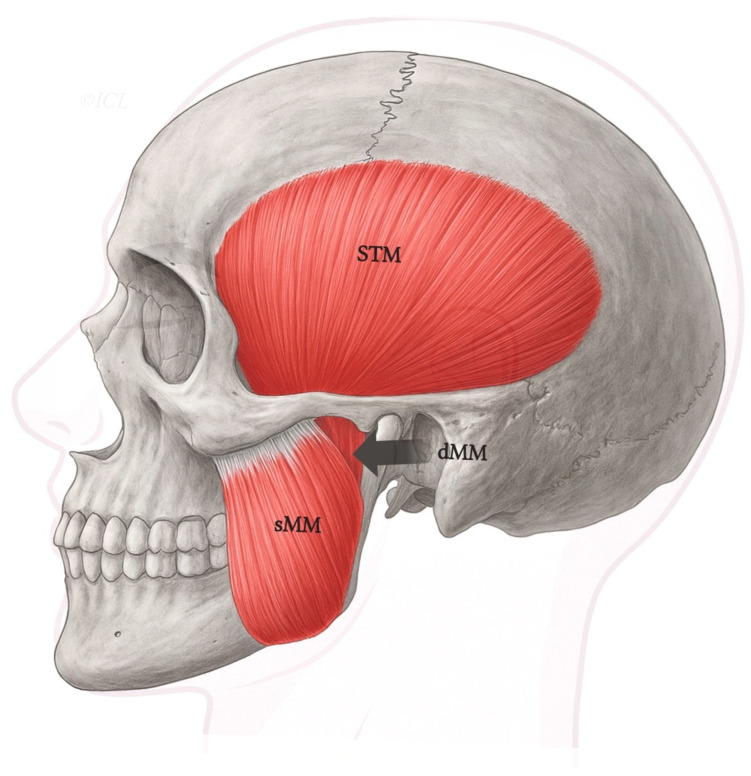
Schematic lateral view of the human masticatory muscles, including the superficial (sMM) and deep (dMM) heads of the masseter, and the temporalis (STM). *Legend:* The deep head (dMM) arises from the medial surface of the posterior zygomatic arch and courses vertically to insert along the superior ramus and coronoid process. In contrast, the superficial masseter (sMM) originates more anteriorly and inserts on the mandibular angle. This classical organization corresponds to Type I in the proposed classification.

Notably, while this binary division is widely accepted, it does not account for a range of observed anatomical variants including accessory bellies, multilayered subdivisions, or neurovascular penetrations which have been sporadically described in the literature but never classified systematically [16,18]. These limitations underscore the need for a more refined morphological classification, which will be addressed in the following sections. The classical spatial organization of the superficial and deep heads of the masseter is schematically illustrated in Figure 1, highlighting the differential origins, fiber orientation, and insertional patterns.

A summary of the key anatomical and functional differences between the SHMM and DHMM is presented in Table 1.

### 4.2. Review of Reported Anatomical Variants of the Deep Head of the Masseter

Although the classical division of the MM into SHMM and DHMM remains foundational, a growing number of anatomical studies have reported notable morphological variations within the deep portion, particularly in terms of its fiber orientation, compartmentalization, neurovascular relationships, and fusion patterns with adjacent muscles.

Several authors have described the presence of accessory bellies or additional muscular slips originating from the posterior zygomatic arch and inserting variably along the mandibular ramus or the coronoid process [4,16]. In some cases, these bellies course medially to the main DHMM and resemble intermediate layers between the deep masseter and the medial pterygoid. Such findings support the hypothesis of a morphological continuum within the pterygomasseteric sling.

Another frequently observed variant includes neurovascular penetrations, particularly by the masseteric nerve or artery. Borschel et al. [18] documented cases in which the masseteric neurovascular bundle perforates the DHMM obliquely, raising important considerations for surgical interventions in this region. Similar penetrations by aberrant branches of the facial artery have been reported in imaging and cadaveric studies [17,19,23,24].

Muscle fiber fusion between the DHMM and the medial pterygoid muscle has also been reported. While some of these fusions are histologically distinct, others demonstrate a true lack of fascial separation, resulting in a blended muscle mass occupying both lateral and medial regions of the Ramus [3]. In rare cases, accessory slips from the DHMM were found to insert onto the temporalis tendon or buccinator fascia, suggesting unusual developmental trajectories [5,19,22,25].

Despite these isolated reports, the literature lacks a unified framework for categorizing these variations. Existing descriptions remain fragmented and terminology inconsistent, with terms such as “accessory deep belly”, “third layer”, or “intermediate lamina” used interchangeably [1,5]. This conceptual gap highlights the need for a clear morphological classification system, which will be proposed in the subsequent Section 4.3.

### 4.3. Proposed Morphological Classification of the Deep Head of the Masseter

In response to the lack of a standardized framework for describing anatomical variations of the DHMM, we propose a novel morphological classification based on cadaveric dissection findings and literature review. This classification includes three main types (Type I–III), reflecting structural differences that may impact clinical and surgical management. Each type is further divided into two subtypes:

- Subtype a—without neurovascular penetration.
- Subtype b—with neurovascular penetration by branches of the masseteric or buccal nerves, which may have implications for botulinum toxin injection, nerve preservation during facial surgery, and electromyographic interpretation (Borschel et al., 2012; Hwang et al., 2005) [17,18].

**Type I—Classical Deep Head (with Fascial Separation)**

**Figure 2:**
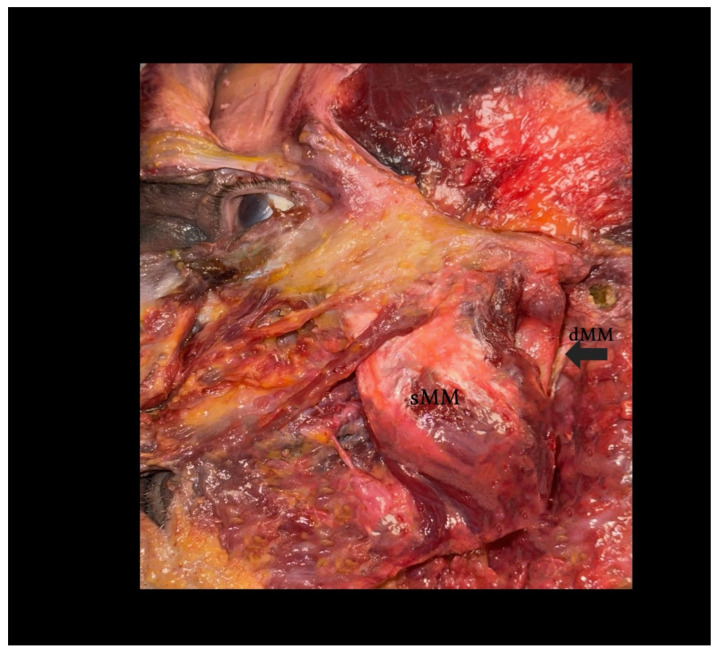
Dissection of the human masseter muscle demonstrating a Type I configuration of the deep head (dMM). *Legend:* The superficial head of the masseter (sMM) has been partially reflected, exposing the vertically oriented fibers of the deep head (dMM), separated by a distinct fascial plane. This classical arrangement allows for clear identification and surgical dissection. Note the dense structure and more posterior–medial location of the dMM relative to the sMM.

This type represents the most commonly described configuration in anatomical textbooks. The DHMM is clearly separated from the superficial portion by a fascial lamina and originates from the posterior and medial surface of the zygomatic arch. It inserts on the upper half of the mandibular ramus or coronoid process and is well-defined, with no fiber blending with adjacent muscles [3,26]—Figure 2.

- Subtype Ia—without neurovascular penetration.
- Subtype Ib—with branches of the masseteric or buccal nerves traversing the muscle belly, visible on high-resolution MRI or Doppler US.

**Type II—Deep Head with Secondary Fusion to the Medial Pterygoid**

In this variant, the DHMM partially fuses with the medial pterygoid muscle, typically at its inferior or posterior aspect. The fusion may range from fascial adherence to complete fiber continuity. This configuration may reflect a developmental anomaly or a retained embryological connection between the two muscles [3,16].

- Subtype IIa—without neurovascular penetration.
- Subtype IIb—with neurovascular penetration by branches of the masseteric or buccal nerves, increasing the risk of nerve injury during deep dissection.

**Type III—Deep Head with Dual Bellies (Segmented Morphology)**

This pattern is characterized by a bifurcated DHMM consisting of two distinct muscular bellies. These may either arise separately from the zygomatic arch or split distally before insertion. The bellies may demonstrate asymmetric fiber orientation or independent neurovascular entry points [4].

- Subtype IIIa—without neurovascular penetration.
- Subtype IIIb—with neurovascular penetration, which may complicate imaging interpretation and surgical planning.

### 4.4. Suggested Terminology

To ensure clarity and consistency in anatomical and clinical communication, we recommend referring to the deep portion of the masseter simply as the “DHMM,” followed by its morphological type (I–III) and subtype (a: without neurovascular penetration; b: with neurovascular penetration) according to the proposed classification.

**Complete list of types and subtypes:**

- DHMM, Type Ia—classical form with fascial separation; no neurovascular penetration.
- DHMM, Type Ib—classical form with fascial separation; with neurovascular penetration.
- DHMM, Type IIa—fused with the medial pterygoid; no neurovascular penetration.
- DHMM, Type IIb—fused with the medial pterygoid; with neurovascular penetration.
- DHMM, Type IIIa—segmented into two muscular bellies; no neurovascular penetration.
- DHMM, Type IIIb—segmented into two muscular bellies; with neurovascular penetration.

This structured terminology avoids ambiguity and promotes ease of use in surgical documentation, imaging reports, and anatomical instruction. Its adoption may improve interdisciplinary understanding and help standardize future publications and atlases [2,16].

## 5. Imaging Characteristics

Accurate identification of the DHMM requires high-resolution imaging techniques that can differentiate between the superficial and deep layers of the masseter, as well as between adjacent structures such as the medial pterygoid, parotid gland, and mandibular ramus. Despite the clinical importance of the DHMM, most radiological protocols do not explicitly address its visualization, leading to potential misinterpretation. In this section, we provide an overview of imaging modalities useful in identifying the DHMM and their strengths and limitations.

### 5.1. Magnetic Resonance Imaging (MRI)

MRI is considered the gold standard for evaluating soft tissue structures in the head and neck due to its excellent tissue contrast resolution. The SHMM and DHMM can often be distinguished based on signal intensity differences and fascial boundaries, especially in high-resolution T1- and T2-weighted sequences [7]. The SHMM typically appears as a homogeneous structure with intermediate signal intensity on both T1 and T2, while the deep head may show slightly lower signal intensity due to its denser, vertically oriented fibers.

The use of fat suppression techniques such as short tau inversion recovery (STIR) or spectral fat saturation (SPIR/SPAIR) enhances contrast between the fascial planes and the muscular tissue, particularly in coronal and axial views. In individuals with distinct Type I anatomy (Type Ia or Ib, i.e., fascial separation, with or without neurovascular penetration), a hypointense linear plane is visible between the two heads, confirming the anatomical boundary. However, in Types II and III (including both subtypes a and b), this fascial delineation may be absent or blurred, complicating identification.

Contrast-enhanced MRI can be particularly useful in differentiating DHMM from pathological masses such as hemangiomas, schwannomas, or lymphadenopathy [27]. Furthermore, volumetric MRI assessments have been used to track changes in the masseter following orthognathic surgery or botulinum toxin injection [25,28].

### 5.2. Diffusion Tensor Imaging (DTI) and Tractography

DTI and MR tractography provide valuable information on the orientation and integrity of muscle fiber tracts. These advanced techniques are especially helpful in assessing the internal architecture of complex muscle layers such as the DHMM, where conventional MRI may fall short [29]. DTI sequences allow visualization of the anisotropic diffusion of water along muscle fibers, enabling 3D tract reconstruction.

In cadaveric studies and in vivo applications, DTI has demonstrated the capability to differentiate between overlapping muscular bundles by fiber orientation. The DHMM typically exhibits more vertical diffusion tracts, while the SHMM shows inferoposterior orientation. In Type III variants (with two bellies), DTI can visualize divergent tracts from separate origins or insertion points.

Although not yet routinely used in clinical practice for masticatory muscle analysis, DTI shows promise in mapping neuromuscular anatomy in preoperative settings, particularly for complex reconstructive procedures or facial nerve transfer planning [30,31].

### 5.3. High-Resolution Ultrasonography (US)

US imaging of the MM is a non-invasive, real-time technique widely used in clinical and aesthetic practice. Modern high-frequency linear probes (12–18 MHz) allow detailed evaluation of the muscle’s SHMM and DHMM layers. The DHMM can often be identified as **a** hypoechoic, vertically aligned structure located deep and posterior to the echogenic fascia of the superficial masseter [32,33].

In patients with well-developed DHMM (Type I or III, subtypes a or b), a hyperechoic fascia may be visible separating the two layers, especially in transverse scans at the mandibular angle. However, in fused variants (Type IIa or IIb), the fascial plane is either poorly defined or absent, requiring dynamic compression and Doppler techniques to distinguish muscle from adjacent vascular structures [8,34].

Ultrasound is particularly valuable for the following:

- Pre-injection mapping in botulinum toxin procedures [10,35];
- Monitoring atrophy or hypertrophy post-surgical or pharmacologic intervention [36];
- Measuring symmetry and muscle thickness in orthognathic and bruxism-related evaluations [29,37].

Despite its operator dependence, US remains the most accessible tool for superficial DHMM evaluation in outpatient settings.

### 5.4. Computed Tomography (CT), MPR, and 3D Reconstruction

Although not optimal for soft tissue contrast, CT imaging remains useful in specific scenarios, particularly when assessing the DHMM in relation to osseous structures or pathology. With multiplanar reconstruction (MPR) and 3D rendering, CT can help differentiate muscle hypertrophy from bony deformities or soft tissue masses [7].

In cases of benign or malignant tumors involving the masticatory space (e.g., intramuscular hemangiomas, parotid tumors with muscular invasion), CT can reveal altered muscle contours, density heterogeneity, or mass effects that may be associated with or mimic DHMM variants.

Furthermore, CT is instrumental in the following:

- Preoperative planning for mandibular osteotomies and angle reduction procedures;
- Detecting calcifications or phleboliths within the muscle [38];
- Assessing symmetry and volume in patients undergoing orthognathic or cosmetic surgery [39].

When used in conjunction with MRI or US, CT adds structural and spatial context that enhances the overall diagnostic accuracy.

## 6. Clinical and Surgical Implications of Deep Head Morphology

### 6.1. Surgical Implications: Orthognathic and Oncologic Perspectives

The DHMM, although frequently underappreciated in surgical protocols, plays a pivotal role in shaping both the technical landscape and risk stratification of orthognathic and oncologic procedures. As outlined in Section 1.3, the DHMM contributes significantly to mandibular stabilization and vertical bite force, and its morphological variability directly impacts dissection, resection, and reconstruction strategies.

#### 6.1.1. Orthognathic Surgery and Mandibular Contouring

In procedures such as mandibular angle osteotomies, ramus contouring, and vertical ramus osteotomy (VRO), the MM be carefully elevated from its bony attachment. A well-developed or hypertrophic DHMM can extend more medially and posteriorly than anticipated, increasing the risk of iatrogenic injury to adjacent neurovascular structures, particularly in cases involving Types IIa/IIb and IIIa/IIIb variants [17,19,23,24].

Angle reduction surgeries, frequently performed for aesthetic refinement, may require partial DHMM resection. However, inadequate management can result in residual bulk or asymmetry, while over-resection can compromise function and lead to facial hollowing [28,40]. MRI and US are essential for preoperative assessment, particularly to identify high-risk configurations such as subtypes Ib, IIb, and IIIb, where neurovascular structures may traverse the deep belly [8,18].

Integration of the proposed DHMM classification into surgical imaging protocols facilitates safer flap planning, nerve-sparing dissection, and improved cosmetic outcomes in mandibular surgery.

#### 6.1.2. Oncologic Considerations and Tumor Mimicry

The DHMM’s proximity to critical anatomical structures in the posterior masticatory space and its variable morphology make it a potential source of diagnostic confusion in oncologic imaging. Variants such as hypertrophic segments, fused layers (Type II), or bifid configurations (Type III) may mimic neoplastic masses, including the following:

- Intramuscular hemangiomas;
- Unilateral masseteric hypertrophy;
- Deep lobe parotid tumors;
- Schwannomas, lipomas, or fibromatosis.

The absence of a distinct fascial plane, especially in Type II variants, complicates differentiation from pathology. Contrast-enhanced MRI and T2-weighted imaging can help distinguish homogeneous DHMM enhancement from irregular tumor signatures [7,27]. Awareness of DHMM types can prevent unnecessary biopsy or surgical overreach.

In oncologic resections, particularly of parotid malignancies, inadvertent DHMM violation may impair postoperative mastication and symmetry. Radiologists and tumor boards should apply the DHMM classification (see Table 2) to improve diagnostic specificity and guide intervention strategies in the masseteric–parotid axis.

### 6.2. Aesthetic, Neuromuscular, and Risk Perspectives

The DHMM is increasingly recognized as a critical structure across diverse clinical domains including aesthetic medicine, neuromodulation, and surgical decision-making. As noted in Section 1.3, its biomechanical function and deep anatomical positioning significantly influence facial contour, bruxism physiology, and iatrogenic risk.

#### 6.2.1. Aesthetic Medicine and Botulinum Toxin Strategies

Botulinum toxin type A (BoNT-A) injection into the masseter is a common aesthetic intervention. However, DHMM variants, particularly Types Ia/Ib and IIIa/IIIb, can result in asymmetric outcomes, suboptimal volume reduction, or unintended complications such as facial hollowing or nerve involvement [8,9,25]. Standard superficial injections may be insufficient in cases with dominant deep volume.

US-guided, anatomy-based injection protocols are recommended. Techniques tailored to DHMM morphology improve treatment precision and reduce side effects, particularly in segmented or neurovascularly penetrated variants [40,41]. MRI volumetry and 3D facial scanning further enhance pre-procedural planning.

#### 6.2.2. Neuromodulation and Electromyographic Applications

The DHMM also plays a role in the management of functional disorders such as bruxism and temporomandibular dysfunction. Variants may influence bite force mechanics, occlusal dynamics, and muscle recruitment patterns [36,42].

Surface EMG primarily reflects superficial fibers, while intramuscular EMG offers deeper diagnostic insights. Inaccurate electrode placement without knowledge of DHMM morphology may yield misleading results. Subtype b (Ib, IIb, IIIb) variants, where nerves penetrate the muscle belly, increase the risk of aberrant neuromodulation or facial weakness following BoNT-A therapy [17].

Proprioceptive features of the DHMM including spindle density may also affect jaw posture and TMD pathophysiology [43]. Thus, identification of DHMM variants should be integrated into diagnostic and biofeedback protocols.

#### 6.2.3. Surgical Risk Stratification and Clinical Planning

Understanding DHMM morphology is essential for tailoring the surgical strategy. Based on the updated classification with subtypes *a* (without neurovascular penetration) and *b* (with neurovascular penetration), we outline a revised risk framework (see Table 3):

- Type I (Classical)—Generally low risk due to clear fascial separation and presents predictable dissection. Subtype Ib presents additional caution because of neurovascular penetration, increasing the likelihood of nerve injury during injections or surgery.
- Type II (Fused with Medial Pterygoid)—Moderate to high risk owing to fusion and loss of fascial boundary. Subtype IIb carries an elevated risk of nerve injury, bleeding, and postoperative deficits due to neurovascular penetration.
- Type III (Dual Bellies)—High risk of tumor mimicry and imaging misinterpretation. Subtype IIIb represents the highest surgical risk, with both mimicry potential and neurovascular penetration, posing significant challenges in BoNT-A injection, resection, or nerve harvesting.

This framework is particularly relevant in facial reanimation procedures, where the masseteric nerve may be embedded within neurovascular subtypes (*b*), complicating harvesting and influencing reinnervation success [31,44]. Routine preoperative assignment of DHMM type and subtype via MRI or intraoperative nerve mapping can aid surgical checklists, enhance training, and mitigate intraoperative risks. These recommendations are based on anatomical synthesis and expert consensus and should be considered provisional until validated through controlled clinical studies.

Table 3 illustrates the risk stratification framework based on the three proposed morphological types of the deep head of the masseter.

Definitive quantification of DHMM’s independent biomechanical effect awaits prospective fine-wire EMG studies and multimodal imaging.

### 6.3. Clinical Takeaways: A Classification-Guided Surgical Perspective

#### 6.3.1. Key Practical Points for Clinicians and Surgeons

- DHMM morphology should always be assessed on preoperative MRI or US in cases involving orthognathic surgery, parotidectomy, or masseter-targeted botulinum toxin therapy.
- Subtypes Ib, IIb, and IIIb (with neurovascular penetration) are associated with increased surgical risk due to the presence of masseteric or buccal nerve branches within the muscle belly.
- Segmented Type III variants (IIIa/IIIb) may mimic tumors or nodular hypertrophy on imaging, leading to diagnostic confusion or unnecessary biopsy.
- In aesthetic procedures, blind superficial injections may result in treatment failure if DHMM volume is dominant. US guidance should be used to target deeper fibers safely, especially in neurovascular subtypes.
- Facial reanimation using the masseteric nerve may be complicated by deep penetration patterns (subtypes b); preoperative imaging and intraoperative nerve mapping are recommended.
- Incorporating the proposed classification system into radiological and surgical planning may improve procedural safety, accuracy, and outcomes.

#### 6.3.2. Clinical Application Box: Practical Use of the DHMM Classification

**Type I (Classical)**

- *Ia*—Ideal for standard surgical planning; low procedural risk; easily visualized on MRI/US.
- *Ib*—As above, but with neurovascular penetration; increased caution during injections or surgery; MRI + Doppler US recommended.

**Type II (Fused with Medial Pterygoid)**

- *IIa*—Risk of misidentification during deep dissection; avoid blind resections; use MRI with fascial plane assessment; may mimic invasive pathology on imaging.
- *IIb*—As above, but with neurovascular penetration; higher risk of nerve injury and bleeding; nerve mapping advised in surgery.

**Type III (Dual Bellies)**

- *IIIa*—Common source of imaging confusion with mass-like lesions; biopsy caution recommended; correlate US/MRI before surgery.
- *IIIb*—As above, but with neurovascular penetration; very high iatrogenic risk in BoNT-A injection or resection; MRI + Doppler US and nerve mapping strongly advised.

## 7. Future Directions

The proposed classification of the DHMM opens several novel avenues for anatomical, radiological, and clinical exploration. Although the superficial portion of the MM has been the focus of the majority of research and intervention strategies, it is becoming increasingly clear that the DHMM, particularly in its variable forms, demands a more central role in future studies on craniofacial surgery, neuromuscular disorders, and aesthetic medicine.

### 7.1. Radiological Standardization and Imaging Protocols

As previously discussed in Section 4, MRI, DTI tractography, and ultrasound elastography provide the highest diagnostic precision for evaluating DHMM morphology. Future research should focus on systematic in vivo validation of these modalities, particularly in the context of prospective studies involving both asymptomatic individuals and patients undergoing facial interventions.

The integration of advanced 3D segmentation techniques, especially those utilizing AI-assisted recognition, may enable automatic identification and classification of DHMM variants (see Table 2), thereby facilitating real-time risk stratification and procedural planning during orthognathic or cosmetic procedures. In addition to MRI and tractography, high-resolution ultrasonography (HRUS) should be emphasized as a valuable, accessible, and cost-effective modality for the evaluation of DHMM morphology in vivo. HRUS enables dynamic assessment, detection of neurovascular penetration, and volumetric follow-up in both clinical and research settings, making it suitable for large-scale, multicenter studies.

### 7.2. Histological and Embryological Investigations

While ontogenetic hypotheses have been proposed—suggesting derivation from the first pharyngeal arch or partial homology with the medial pterygoid muscle—a definitive embryological origin of the DHMM remains unresolved. Histochemical and immunohistochemical profiling, including markers of muscle fiber type, proprioceptive structures (e.g., muscle spindles), and neural crest contributions, may shed light on the developmental divergence of this structure and its functional compartmentalization. Furthermore, such studies could clarify why certain variants (e.g., Types III and subtypes b configurations) are associated with specific clinical syndromes or complications.

### 7.3. Clinical Trials and Procedure-Specific Algorithms

Given the strong correlation between DHMM variants and procedural outcomes, interventional trials incorporating anatomical classification as a covariate are warranted. These include the following:

- Botox trials with stratified injection protocols based on DHMM morphology;
- Orthognathic and contouring surgery with intraoperative classification-based adjustments;
- TMD treatments considering deep head asymmetry or overuse.

Future clinical guidelines may include the DHMM subtype as a standard preoperative descriptor, similar to the use of vascular or nerve variant reporting in neurosurgery or hepatobiliary surgery. Future research should also include structured inter-rater reliability studies, multicenter reproducibility assessments, and prospective trials correlating DHMM subtypes with surgical and functional outcomes. Such evidence will be critical to confirm the practical validity of the proposed taxonomy.

### 7.4. Surgical Training and Cadaveric Dissection Curricula

Medical education and surgical training in maxillofacial anatomy should expand beyond the traditional superficial–deep dichotomy. The three-type model introduced in this review may serve as a didactic framework for cadaver-based training, particularly in centers offering maxillofacial fellowships. Inclusion of variant-specific dissection protocols, reinforced with US or MRI correlation, could elevate anatomical literacy and reduce procedural errors.

### 7.5. Integration into Official Anatomical Terminology Systems

Currently, the DHMM is not listed as a separate entry in major anatomical ontologies, such as *Terminologia Anatomica* or the *Foundational Model of Anatomy.* It is typically subsumed under the general heading of the MM, without acknowledgement of its structural and clinical distinctiveness. Given its complex morphology, variable presentation, and increasing relevance in diagnostic imaging, reconstructive surgery, and aesthetic interventions, we strongly advocate for the formal inclusion of the DHMM as defined by the proposed classification into international anatomical terminological systems. This would support standardization in academic curricula, research databases, and clinical documentation.

Standardized imaging and reporting could enable pooled analyses of DHMM morphology; prospective multicenter protocols are needed before any decision-support tools are considered.

## 8. Limitations

While the present review provides a comprehensive anatomical, radiological, and clinical analysis of the DHMM, several limitations must be acknowledged, particularly regarding evidence heterogeneity, sample availability, and methodological constraints.

### 8.1. Heterogeneity of Anatomical Descriptions

One of the main challenges encountered during this review was the inconsistency in anatomical terminology and reporting across the literature. Many studies either conflate the DHMM with the SHMM or fail to distinguish it from adjacent structures such as the medial pterygoid or parotid mass. The lack of standardized dissection protocols and terminology impairs direct comparison and limits the robustness of morphotype classification synthesis [3,4].

### 8.2. Limited Sample Size in Cadaveric and Imaging Studies

Despite recent interest in MM morphology, studies specifically addressing the DHMM remain scarce, with small sample sizes and often unilateral dissections. Most cadaveric investigations involve fewer than 30 specimens, and many lack demographic data (e.g., sex, age, ethnicity), which restricts generalizability. Imaging studies, while expanding, often focus on superficial muscle layers or fail to report deep head involvement explicitly.

### 8.3. Radiological Ambiguity and Validation Gaps

Although MRI and high-resolution ultrasound are increasingly used in masticatory muscle evaluation, there remains limited radiological validation of DHMM types and subtypes in vivo. Differentiation between muscle variants and pathological entities (e.g., hemangiomas, fibromas, lipomas) is particularly challenging in atypical configurations such as **Type III** and in *subtypes b* of any type, where neurovascular penetration may alter signal characteristics or contour. Furthermore, there is currently no consensus on imaging protocols or criteria for standardized classification of these variants.

### 8.4. Absence of Prospective Clinical Trials

Most surgical insights included in this review are derived from case reports, retrospective analyses, or expert opinion. There is currently no prospective evidence linking the DHMM subtype to outcomes in orthognathic surgery, botulinum toxin efficacy, or facial reconstruction. As such, the proposed risk stratification framework remains theoretical and requires validation in clinical trials.

### 8.5. Functional and Electrophysiological Data Deficit

Electromyographic and functional studies of the MM rarely isolate the DHMM from the SHMM. As a result, our understanding of its neuromuscular physiology, proprioceptive role, and response to interventions is incomplete. Without this data, it is difficult to correlate morphological types with clinical symptoms (e.g., bruxism, TMD) or predict functional outcomes.

The DHMM represents a clinically relevant anatomical entity whose significance has long been underappreciated in both the classical anatomical literature and surgical practice. Through this review, we have proposed a novel four-type morphological classification that integrates gross anatomy, radiological characteristics, and surgical implications, offering a practical framework for clinicians and researchers alike.

This classification not only improves anatomical understanding but also provides a foundation for risk stratification in facial surgery, particularly in orthognathic interventions, aesthetic procedures, and masseter-targeted therapies. The delineation of DHMM subtypes emphasizes the need for standardized anatomical descriptions and encourages the integration of deep muscular components into surgical checklists and educational modules.

Based on current evidence, MRI and high-resolution US should be considered the first-line modalities for identifying and evaluating DHMM variants preoperatively. These imaging techniques allow for improved diagnostic accuracy, reduction of iatrogenic risks, and informed surgical planning.

Future research should aim to fill existing gaps in histological characterization, immunohistochemical profiling, and functional biomechanical studies, particularly those correlating DHMM morphology with neuromuscular physiology and clinical outcomes. Moreover, clinical case series and interventional trials stratified by DHMM type will be essential to validate the proposed framework and refine clinical guidelines.

Finally, we advocate for the systematic inclusion of the DHMM in standard anatomical atlases, imaging references, and surgical textbooks. Recognition of its distinct morphology, variability, and clinical significance is essential for advancing the field of craniofacial anatomy and ensuring safer, more precise surgical interventions. This review synthesizes heterogeneous cadaveric and imaging reports, many of which involve small sample sizes and inconsistent terminology. As such, the proposed classification should be considered preliminary. No inter-observer reliability assessment, statistical validation, or correlation with clinical outcomes was performed, and these remain essential steps for confirming reproducibility and clinical applicability in future studies. Although the proposed classification draws from heterogeneous sources, every effort was made to harmonize terminology and morphological definitions to reduce the risk of perpetuating inconsistencies. Nonetheless, the framework should be refined through standardized multicenter anatomical and imaging studies. Moreover, no prospective or randomized clinical studies have yet confirmed the association between DHMM subtype and surgical or functional outcomes, and current recommendations should be regarded as preliminary until validated by such evidence.

## 9. Conclusions

The deep head of the masseter muscle (DHMM) is a morphologically and functionally distinct structure whose variability carries significant diagnostic, surgical, and therapeutic implications. The proposed three-type morphological classification, each with two subtypes based on the presence or absence of neurovascular penetration, provides a standardized and clinically oriented framework for anatomical description, radiological identification, and surgical risk stratification. By integrating cadaveric, radiological, and literature-based evidence, this system offers a reproducible nomenclature that can improve interdisciplinary communication and guide preoperative planning, particularly in orthognathic surgery, facial reanimation, temporomandibular joint management, and aesthetic interventions.

Future research should focus on large-scale, multicenter validation of this taxonomy, development of imaging protocols tailored to DHMM subtypes, and incorporation of this terminology into official anatomical standards. Such efforts will bridge the current gap between anatomical variability and precision clinical practice, ultimately enhancing patient safety and surgical outcomes.

## Figures and Tables

**Table 1:** Comparative features of the superficial and deep heads of the masseter muscle.

Feature	Superficial Head	Deep Head
**Origin**	Anterior two-thirds of the zygomatic arch	Posterior third of the zygomatic arch (inner surface)
**Insertion**	Inferior half of mandibular ramus and angle	Superior ramus and sometimes coronoid process
**Fiber Orientation**	Oblique inferoposterior	Vertical
**Function**	Elevation and protrusion	Elevation and mandibular stabilization
**Fascial Separation**	Frequently distinct	Variable; may be absent in fused types
**EMG Profile**	Dominant in light clenching and mastication	Active during forceful biting and bruxism
**Imaging Characteristics**	Homogeneous signal, anterior location	Denser fibers, deeper position, distinguishable on MRI/US
**Clinical Relevance**	Primary target in aesthetic contouring	Key structure in surgical risk and diagnostic confusion

**Table 2:** Proposed morphological classification of the deep head of the masseter muscle.

Type	Description	Subtype	Clinical Implication	Imaging Features
I	Classical deep head, separated by fascia	Ia—without neurovascular penetration	Predictable anatomy; standard surgical planning	Clearly delineated on MRI; hypointense fascial plane on T1/T2
		Ib—with neurovascular penetration	Added caution in injections, flap elevation, or nerve monitoring	As above, plus neurovascular tract visible on high-resolution MRI or Doppler US
II	Deep head partially or fully fused with medial pterygoid	IIa—without neurovascular penetration	Risk of misidentification during deep dissection	Blurred interface with medial pterygoid on MRI or US; indistinct fascial boundary
		IIb—with neurovascular penetration	Increased risk of nerve injury during surgery	As above, plus neurovascular tract visible on high-resolution MRI or Doppler US
III	Segmented deep head with two distinct muscular bellies	IIIa—without neurovascular penetration	Possible confusion with tumor or hypertrophic mass	Bipartite appearance on MRI; possible asymmetric US echotexture
		IIIb—with neurovascular penetration	Added surgical and injection risk	As above, plus neurovascular tract visible on high-resolution MRI or Doppler US

**Table 3:** Surgical risk stratification according to deep head morphology.

Type	Subtype	Anatomical Characteristics	Primary Risk Domains	Surgical Risk Level
I	Ia	Well-separated deep head with clear fascial boundary	Predictable anatomy, safe dissection	Low
	Ib	As Ia, but with neurovascular penetration	Added risk of nerve injury during injections or surgery	Low to Moderate
II	IIa	Fusion with medial pterygoid, fascial boundary absent or incomplete	Misidentification, excessive resection, bleeding	Moderate to High
	IIb	As IIa, but with neurovascular penetration	Increased risk of nerve injury, bleeding, and postoperative deficits	High
III	IIIa	Segmented head with two muscular bellies, atypical insertion	Tumor mimicry, imaging misinterpretation, biopsy risk	High
	IIIb	As IIIa, but with neurovascular penetration	Added risk of nerve injury, injection complications, neuromuscular deficits	Very High
